# Rhabdomyolysis after the free fibular flap operation for mandibular reconstruction: a case report

**DOI:** 10.1186/s40902-018-0180-2

**Published:** 2018-12-14

**Authors:** Won-Hyuk Choi, Yong-Deok Kim, Jae-Min Song, Jae-Yeol Lee

**Affiliations:** Department of Oral and Maxillofacial Surgery, School of Dentistry, Pusan National University, Yangsan-si, Gyeongsangnam-do Republic of Korea

**Keywords:** Free fibular flap, Pneumatic tourniquet, Rhabdomyolysis, Kidney failure

## Abstract

**Background:**

Free fibular flap is one of the most useful methods in the hard tissue reconstruction of the maxilla-mandible. Free fibular flap presents some advantages in which the reconstruction of both soft and hard tissues can be done at the same time. It also provides a safe and successful bone graft for the reconstruction, along with a low rate of complications. Despite these advantages and the rarity of a postoperative complication, particularly in oral and maxillofacial surgery procedures, a prolonged operation might exhibit some complications related with rhabdomyolysis. We experienced the rare event of rhabdomyolysis after oral cancer surgery.

**Case presentation:**

In this article, we report the case of a patient who developed rhabdomyolysis after undergoing free fibular flap surgery.

**Conclusions:**

Despite the advantages of the free fibular flap operation, clinicians must be aware of the risk of complications because there are multiple factors that could result in rhabdomyolysis, such as duration of operation, position of the subject, and pre-existing conditions of diabetes and hypertension. Once the diagnosis of rhabdomyolysis is confirmed, a prompt treatment plan should be made and applied as soon as possible. This will increase the chance of a full recovery for the patient who is exhibiting symptoms of rhabdomyolysis.

## Background

Free fibular flap is one of the most useful methods in the hard tissue reconstruction of maxilla-mandible. It also provides a safe and successful bone graft for the reconstruction, along with a low rate of complication [1]. Despite these advantages and the rarity of a postoperative complication, particularly in oral and maxillofacial surgery procedures, a prolonged operation might exhibit some complications that are related to rhabdomyolysis. Clinicians must be aware of certain risk factors, and the strict guidelines for mid-application release and inflation pressure must be followed in a timely manner to prevent rhabdomyolysis [2]. We experienced the rare occurrence of rhabdomyolysis after oral cancer surgery.

Rhabdomyolysis is a severe syndrome characterized by the destruction of the skeletal muscle cells, leading to the leakage of muscle components into the blood vascular system [3]. Its symptoms depend on the severity of kidney failure, which may include muscle pain, weakness, vomiting, and confusion. When the condition of rhabdomyolysis is mild, muscle symptoms may not be detected, and abnormal blood tests in the context of other problems might be used for the diagnosis. A more severe form is characterized by muscle pain, tenderness, weakness, and swelling of the affected muscles [3–7].

In this article, we report the case of a patient who developed rhabdomyolysis after oral cancer ablation surgery and reconstruction with fibula-free flap. The patient was diagnosed with squamous cell carcinoma on the right buccal mucosa of the mandible and underwent surgical procedures involving the resection and reconstruction of the site. We were able to detect early symptoms of rhabdomyolysis 1 day after the surgery. An effective treatment was applied immediately, and the patient fully recovered from the early stages of rhabdomyolysis. Additionally, there are some literatures that provide evidence on our treatment that was applied to the patient.

## Case presentation

A 74-year-old woman visited our station with squamous cell carcinoma (SCC) on the right buccal mucosa. Her past medical history included chronic obstructive airways disease, hypertension, and diabetes mellitus. The patient is a current smoker, with a history of 20 pack-years. Preoperative chest radiography, electrocardiogram, full blood count, and serum biochemistry were within the normal range. After being diagnosed with SCC as a result of incisional biopsy, the patient underwent the resection of SCC on the right buccal mucosa of the mandible, modified radical neck dissection, and primary reconstruction with a fibula-free flap. Tourniquet pressure was 300 mm/Hg, and its application time was 60 min. Total on-table time was approximately 7 h. Upon admission to the SICU after the 7-h operation, hypothermia and hypotension were noted. On the first postoperative day, the patient exhibited oliguria and proteinuria and elevation of CK, AST, ALT, and LDH. Together with the nephrology and neurology staff, we tried to figure out our patient’s symptoms and clinical findings. We thought that her clinical picture was based on an impression in which acute renal failure was diagnosed as secondary to rhabdomyolysis. Thus, she was managed with high-dose loop diuretic therapy. Additionally, we gave her hepatotonic to recover her liver function. The patient was supplemented with 150 to 250 mL/h of lactated Ringer’s solution and 0.9% NaCl. When the volume was full, urine output of above 100 mL/h was maintained by 20 mg intravenous injection with furosemide.

**Fig. 1:**
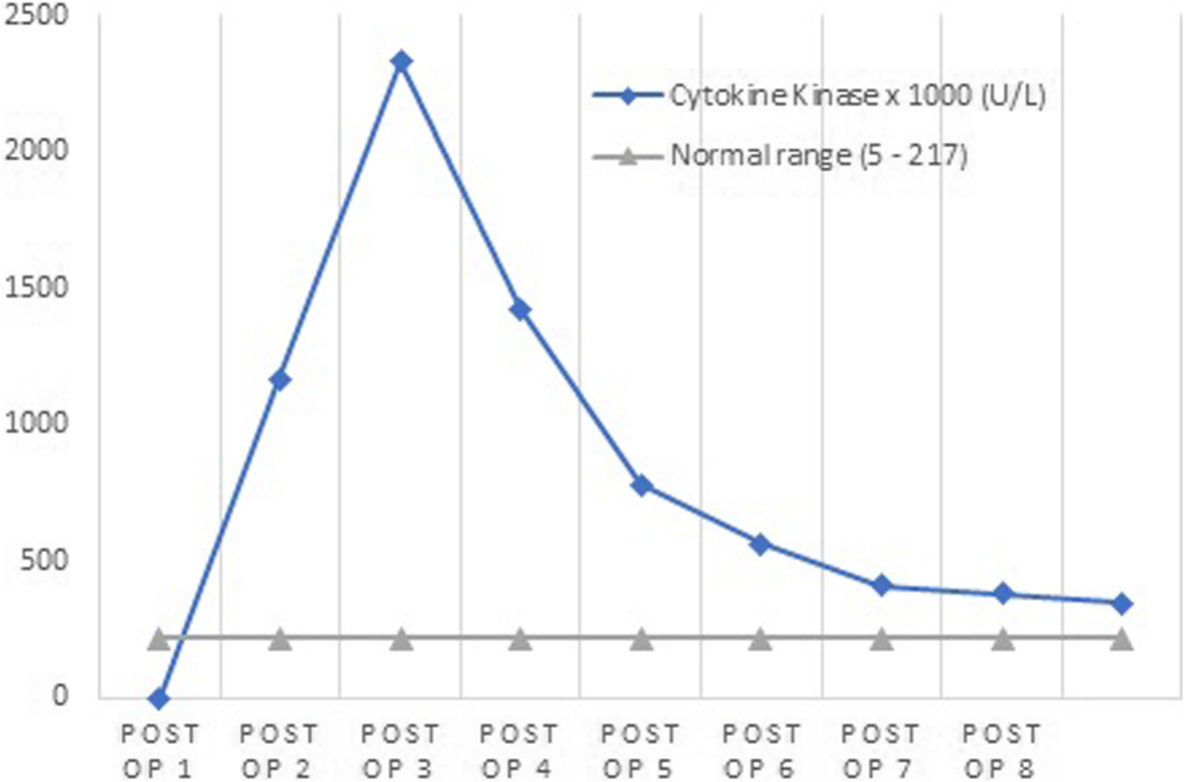
The changing levels of serum creatinine kinase (U/L) after the operation. CK value sharply increased 2 days after operation followed by a dramatic escalation on the third day postoperation. However, as swift and timely diagnosis and treatment was performed, 5 days after the operation, the value of CK dropped to normal range. CK value on the figure displayed as 1000 times. Abbreviations: CK, creatinine kinase, Post-op, postoperative day

**Fig. 2:**
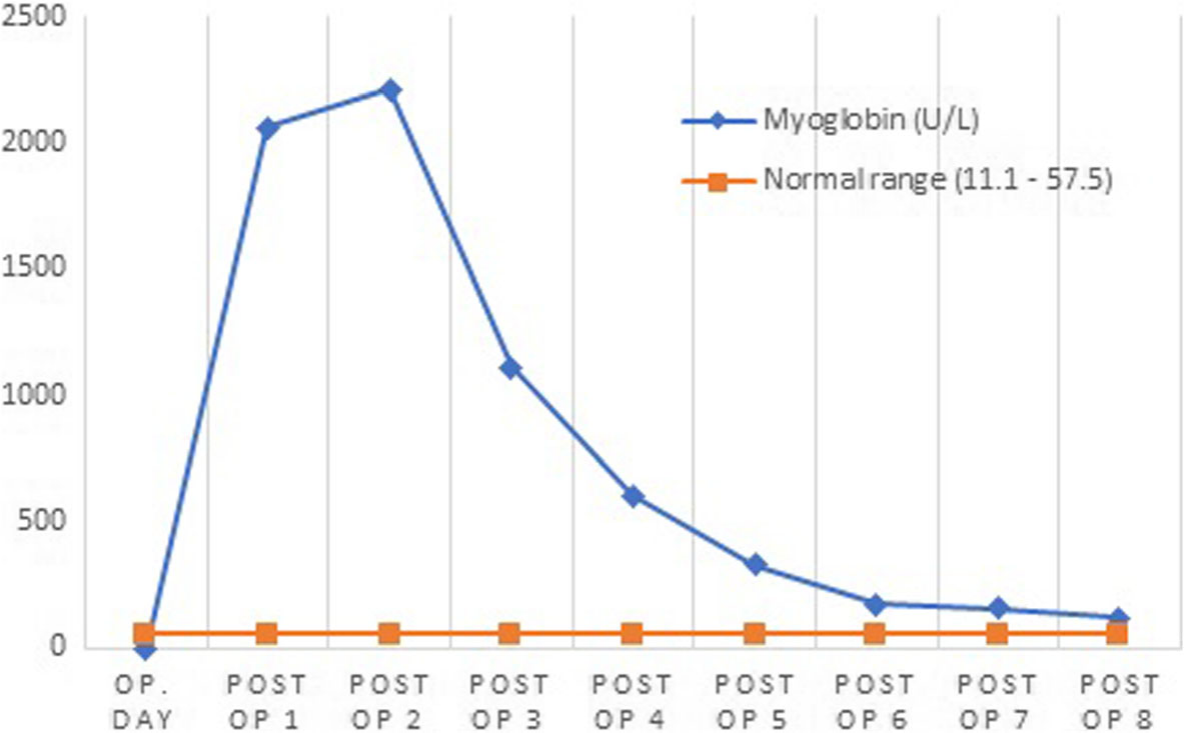
The changing levels of serum myoglobin (U/L) after the operation. Serum myoglobin value sharply increased 2 days after operation followed by a dramatic escalation on the third day postoperation. However, as swift and timely diagnosis and treatment was performed, 5 days after the operation, the value of CK dropped to normal range. Abbreviations: Op., operation, Post-op, postoperative day

Her urine output for the first hour is at 20 mL/hour, but after the medication, her urine output began to improve on day 4 with a corresponding reversal in the serum creatinine. After postoperative day 4, the muscular enzyme showed a downward trend. We treated the patient with medication and hydration, and then the result became favorable. In the end, she was able to recover fully from the symptoms. Figures 1 and 2 show the change in serum enzyme levels during hospitalization (Figs. 1 and 2).

## Discussion

**Fig. 3:**
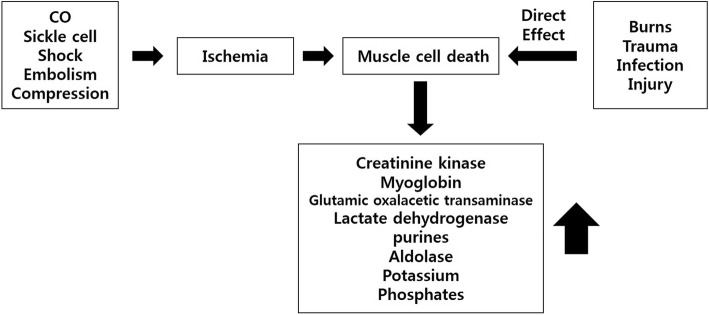
Mechanisms of rhabdomyolysis

Rhabdomyolysis is a spectrum of the clinical picture due to the damage of myocytes and the leakage of cellular materials to the vascular stream. The clinical feature of rhabdomyolysis varies from the asymptomatic elevation of the liver functional lab result to motility, electrolyte imbalance, and acute kidney injury [8, 9]. Postoperative rhabdomyolysis is associated with several factors: non-supine position (prone, lateral, and variations of lithotomy) during surgery, prolonged surgery time, and use of tourniquet during surgery [10]. Figure 3 describes the mechanisms of rhabdomyolysis. (Fig. 3).

If there is no suspicion, the diagnosis of rhabdomyolysis may be missed because myalgia and swelling may not be observed. Therefore, the laboratory test result, such as CK and urine myoglobin, should be confirmed for the definitive diagnosis of rhabdomyolysis. Additionally, biopsy may be done to ascertain the diagnosis. There are no specific diagnosis criteria in rhabdomyolysis, but many clinicians determine diagnosis when a serum enzyme, such as CK and myoglobin, is five times higher than the normal range [11].

Although a rare complication of the operation, especially in maxillofacial surgery, postoperative rhabdomyolysis has been reported in several surgical fields, including urology, neurosurgery, and orthopedic, cardiovascular, and bariatric surgeries. Several risk factors have been identified, including long-term surgery (> 7 h) without proper repositioning, location of the patient (generally crush or side pressure sores), extended tourniquet time (> 1 h), and other systemic complications, such as diabetes, hypertension, and peripheral vascular disease [8–10, 12–15].

Our patients meet a variety of causes or conditions that contribute to the development of rhabdomyolysis, such as diabetes, hypertension, use of pneumatic tourniquets, dehydration, and prolonged surgery. However, in this patient’s case, it is very difficult to determine the exact cause of rhabdomyolysis.

No matter what the cause, while under the diagnosis of rhabdomyolysis, hydration therapy should be applied, and toxic enzyme must be removed out of the bloodstream. To avert continuous muscle damage, such as trauma, infection must be determined and managed directly [16]. With early and definitive diagnosis and treatment, it is believed that the patient can have a bright prognosis. Moreover, the recovery of full renal function is also warranted [11]. Additionally, it is recommended that daily routine measurements of liver functional lab, especially CK and myoglobin, be administered [14].

Despite the advantages of the free fibular flap operation, clinicians must be aware of the risk of complications because there are multiple factors that could result in rhabdomyolysis, such as duration of the operation, position of the subject, and existing conditions of diabetes and hypertension. Therefore, close monitoring of certain measures is required, and it is recommended that procedures may be divided into two or three stages to avoid prolonged muscle compression when prolonged surgical time is anticipated. Once a diagnosis of rhabdomyolysis is confirmed, a prompt treatment plan should be made and applied as soon as possible. This will increase the chance of a full recovery for the patient who is exhibiting symptoms of rhabdomyolysis.

## Conclusion

Rhabdomyolysis is a rare complication during free flap surgery operation that may result in renal failure. Once a diagnosis of rhabdomyolysis is confirmed, a proper treatment plan, such as fluid resuscitation, should be made and applied as soon as possible. We have reported a very rare case of rhabdomyolysis after free fibular flap surgery. Thus, clinicians must be aware of the risk of rhabdomyolysis and perform close monitoring.

## Abbreviations

ALT: Alanine aminotransferase
AST: Aspartate aminotransferase
CK: Creatinine kinase
Fig: Figure
LDH: Lactate dehydrogenase
op: Operation
Post-op: Postoperative
SCC: Squamous cell carcinoma
SICU: Surgical Intensive Care Unit
